# Beyond acid suppression: the multifaceted role of proton pump inhibitors in *Helicobacter pylori* eradication

**DOI:** 10.3389/fmicb.2026.1947561

**Published:** 2026-08-24

**Authors:** Paulina Sroczyńska, Paweł Krzyżek

**Affiliations:** 1 Department of Endocrinology, Diabetology and Internal Medicine, T. Marciniak Lower Silesian Specialist Hospital - Emergency Medicine Center, Wrocław, Poland; 2 Department of Microbiology, Faculty of Medicine, Wrocław Medical University, Wrocław, Poland

**Keywords:** antimicrobial activity, eradication therapy, gastric acid, *Helicobacter pylori*, PPIs, proton pump, proton pump inhibitors

## Abstract

Gastric acid is essential for digestion, host defense, and maintenance of gastrointestinal homeostasis; however, its excessive or inappropriate secretion contributes to the development and progression of several acid-related disorders. Proton pump inhibitors (PPIs) have remained the cornerstone of acid-suppressive therapies for more than four decades owing to their potent blockade of gastric H^+^/K^+^-ATPases. This review provides a comprehensive overview of the pharmacological properties, clinical applications, and current limitations of the most widely used PPIs, including omeprazole, esomeprazole, lansoprazole, dexlansoprazole, pantoprazole, and rabeprazole. Particular emphasis is placed on their role in the management of *Helicobacter pylori* infection, a strong risk factor for the development of severe gastric diseases and one of the most clinically important indications for PPI-based antibiotic therapy. Beyond elevating intragastric pH to enhance antibiotic stability and efficacy, accumulating evidence indicates that PPIs exert direct antibacterial activity against *H. pylori* and may act synergistically with selected antibiotics. The review also discusses emerging evidence for interindividual variability in PPI metabolism, drug-drug interactions, and concerns regarding long-term adverse effects of the current treatments. Finally, it highlights potassium-competitive acid blockers (P-CABs), a newer class of acid-suppressive agents that provide sustained acid inhibition and represent a promising alternative to PPI-based regimens for *H. pylori* eradication.

## 1 Gastric acid: clinical significance and development of acid-suppressive therapies

### 1.1 Clinical significance

The gastrointestinal tract is a complex system of organs responsible for digestion, absorption, secretion, and immune regulation. Its walls are composed of four histological layers: the mucosa, submucosa, muscularis externa, and serosa with adventitia. The stomach is a highly specialized digestive organ situated between the esophagus and the duodenum, whose role involves the mechanical and chemical breakdown of food by gastric acid and digestive enzymes. Gastric mucosa contains distinct glandular cells, including parietal, chief, mucous, and enteroendocrine cells, which are involved in hydrochloric acid secretion, enzyme production, and regulation of gastrointestinal physiology (Ogobuiro et al., 2023).

Gastric acid is secreted by parietal stomach cells and is considered a key factor in physiological upper gastrointestinal functioning. The hydrochloric acid keeps the stomach pH value between 1.5 and 2, and therefore functions as a vital antimicrobial barrier. This activity is maintained effectively at pH ≤ 3, whereas reduced gastric acidity creates favorable conditions for the growth of a wide range of microorganisms, increasing the risk of infections caused by *Clostridioides difficile*, *Salmonella* spp., *Shigella* spp., *Campylobacter jejuni*, and pathogenic strains of *Escherichia coli*. Reduced acidity may also lead to quantitative and qualitative changes in the microbial structure of the digestive system and alterations in the gastric microbiota or development of small intestinal bacterial overgrowth (SIBO) (Hunt, 1988; Bavishi and DuPont, 2011).

Considering the multitude of functions performed by the stomach, inappropriate gastric acid secretion is related also to several pathological conditions of non-microbiological origin, including gastroesophageal reflux disease (GERD), gastric ulcers, and ulceration in hypersecretory conditions (such as Zollinger-Ellison syndrome) (Huang and Hunt, 1996; Farley et al., 2000). About sixty years ago, these conditions could be life-threatening if left untreated, as therapeutic options were very limited at that time (Malfertheiner et al., 2009). For example, for peptic ulcers, the main treatment consisted of administration of antacids to neutralize excess gastric acid. Although this approach was favorable, it provided only temporary relief. The alternative was either a gastrectomy or a vagotomy, a procedure in which the nerves supplying the stomach are severed. It should be noted that acid-reducing surgeries carried a significant risk of immediate, intraoperative complications, including death, bleeding, and injury to the stomach or esophagus. Other complications, such as post-operative diarrhea, dumping syndrome or vitamin B_12_ deficiency, were also observed more frequently (Seeras et al., 2022). Pharmacological control of the complex mechanism of gastric acid secretion has therefore long been desirable (Olbe et al., 2003).

### 1.2 Development of acid-suppressive therapies

The medical treatment of acid-related diseases underwent a breakthrough in the late 1970s. During this period, James Black and his team developed cimetidine, the first histamine H_2_-receptor antagonist (H_2_RA). It was introduced into clinical practice in 1976. Then, in the 1980s, additional agents from this class were introduced, including ranitidine (1981) and famotidine (1985) (Janiaczyk and Ogrodowczyk, 2021). H_2_RAs improved the lives of millions of people and have reduced the need for surgery, but have a relatively short duration of action. Independently, from the late 1960s, Astra initiated a research program aimed at finding a drug effectively inhibiting gastric acid secretion. This research ultimately led to the development of proton pump inhibitors (PPIs). Omeprazole, the first PPI implemented into clinical practice, was launched in 1989 under the brand names Losec in Europe and Prilosec in the United States. It was rapidly demonstrated to be more effective than H_2_RAs due to its prolonged duration of acid suppression (Olbe et al., 2003).

Following the introduction of omeprazole, several other PPIs were subsequently developed and introduced into clinical practice, including pantoprazole, esomeprazole, lansoprazole, dexlansoprazole, and rabeprazole (Dominiczak et al., 2024). Further progress in PPI therapy led to the development of a new generation of long-acting agents with prolonged plasma half-life. Currently, ilaprazole is the only marketed representative of this group, while other compounds, such as tenatoprazole, azeloprazole, anaprazole, AGN201904-Z (Durasec™), and DLBS-2411 (Redacid^®^), remain under clinical trials (Hunt et al., 2008; Scarpignato and Hunt, 2008; Hunt and Scarpignato, 2018; Srebro et al., 2022).

Since the introduction of PPIs, these drugs rapidly became the primary treatment for acid-related disorders (Strand et al., 2016). In addition to their prolonged duration of action, PPIs provide superior control of both postprandial and nocturnal gastric acidity, which is clinically relevant in some patients (Howden, 1997). Moreover, the acid-suppressive efficacy of PPIs remains stable during long-term therapy without the need for dose escalation. Unlike H_2_RAs, PPIs are not associated with the development of tachyphylaxis during treatment (Wilder-Smith et al., 1990). These beneficial properties have made PPIs a cornerstone of therapy for acid-related disorders for over 40 years.

## 2 Proton pump inhibitors: chemical structure, mechanism of action, and pharmacological properties

### 2.1 Chemical structure

**FIGURE 1:**
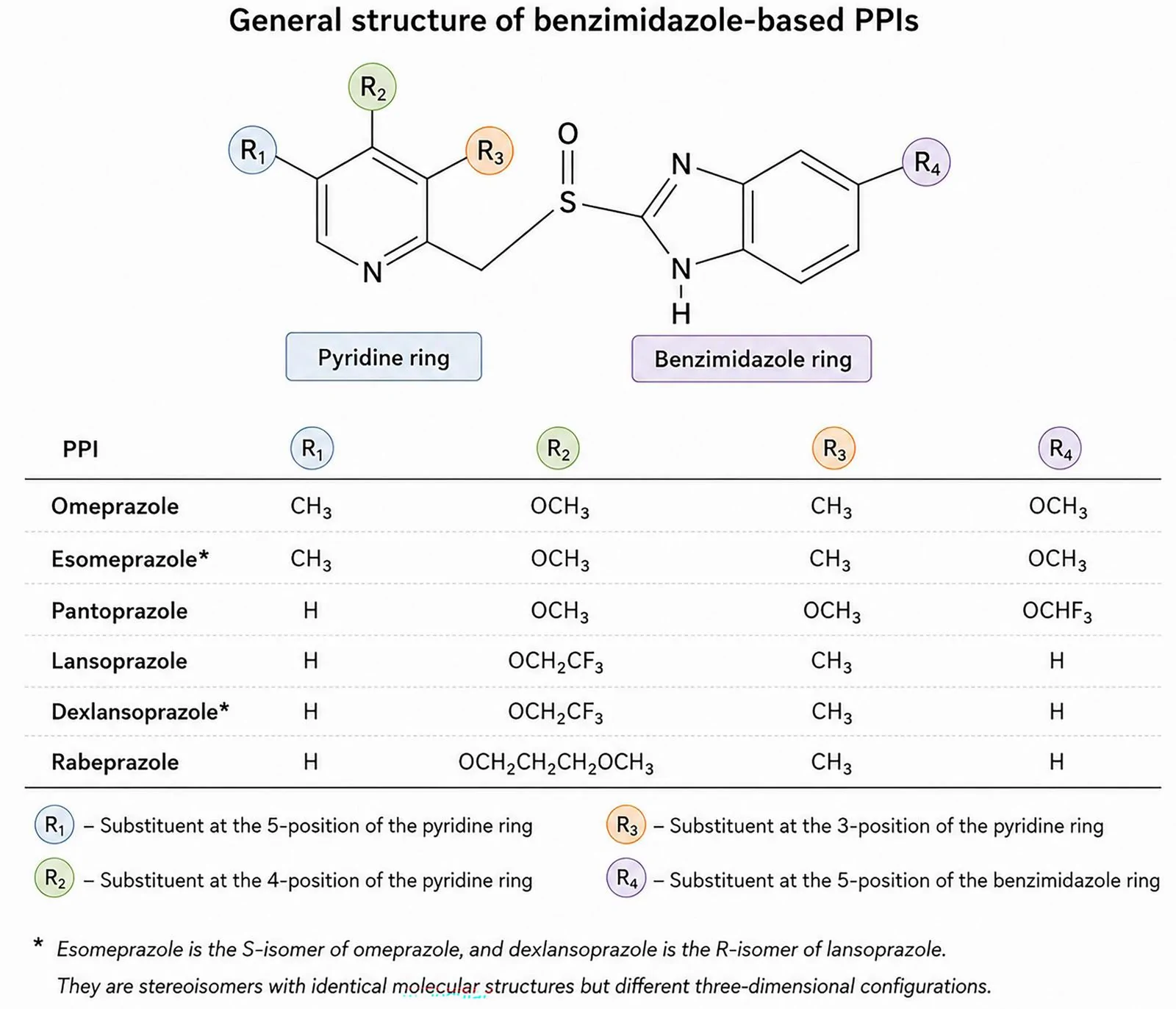
The chemical structure of commonly used proton pump inhibitors (PPIs).

PPIs constitute a relatively small and well-characterized class of drugs. Although there are slight differences in their chemical structures, all compounds within this group share a common mechanism of action and a similar core structure (Srebro et al., 2022). Omeprazole was the first benzimidazole-derived PPI applied in clinical practice. Other benzimidazole PPIs were subsequently introduced, including lansoprazole, pantoprazole, and rabeprazole. The first two compounds are composed of two heterocyclic rings: a pyridine moiety and a benzimidazole moiety connected by a methylsulfinyl group (Sachs, 2003). Rabeprazole differs structurally from other PPIs by the presence of a methoxypropoxy substituent on the pyridine ring, which contributes to its rapid activation and distinct pharmacokinetic profile (Roche, 2006; Bakheit et al., 2021). In contrast to conventional PPIs based on a benzimidazole structure, tenatoprazole contains an imidazopyridine ring (Srebro et al., 2022). Esomeprazole and dexlansoprazole are optical isomers of omeprazole and lansoprazole, respectively. Although both compounds share an identical molecular structure, they differ in their three-dimensional spatial configuration. This stereochemical difference refers to the opposite orientation of substituents around the chiral center, which influence their pharmacokinetic and pharmacodynamic properties despite the same chemical formula (Andersson et al., 2001; Skrzydło-Radomańska and Radwan, 2015). The chemical structures of representative PPIs are shown in Figure 1.

### 2.2 Mechanism of action

**FIGURE 2:**
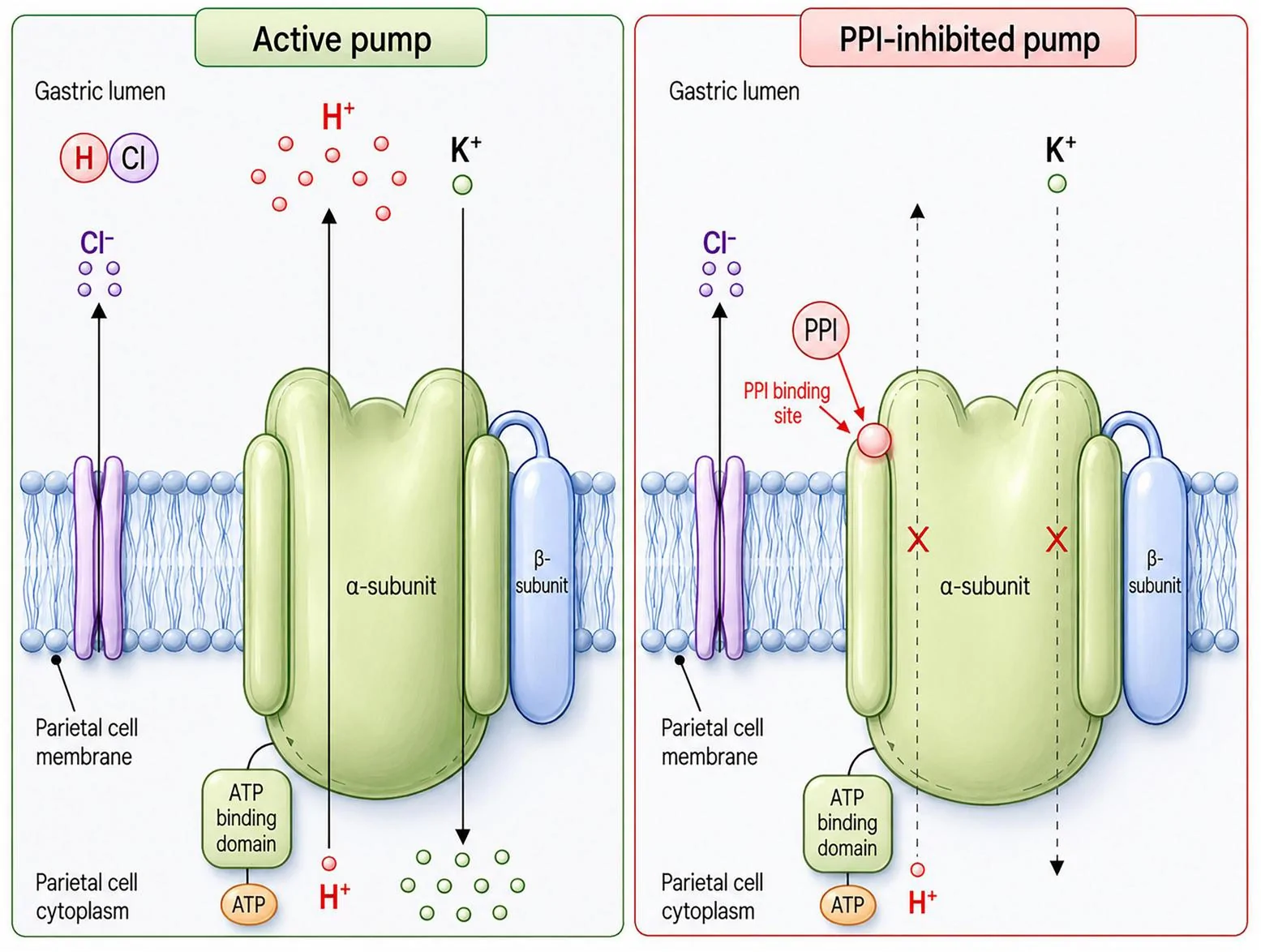
Mechanism illustrating the proton pump inhibitor (PPI)-dependent inhibition of the gastric H^+^/K^+^-ATPase activity.

PPIs are a class of medications that effectively suppress gastric acid secretion through selective inhibition of proton pumps (Fellenius et al., 1981). These pumps are membrane-bound transport enzymes known as H^+^/K^+^-ATPases, located in cytoplasmic membranes of resting parietal cells of the stomach. Gastric H^+^/K^+^-ATPases are heterodimeric membrane enzymes composed of a large catalytic α-subunit and a smaller β-subunit. The α-subunit contains the ATP-binding and phosphorylation domains and is responsible for proton transport across the membrane, whereas the β-subunit is essential for proper folding, membrane targeting, and stabilization of the enzyme complex (Shin et al., 2008). Its primary function is to actively secrete hydrogen ions (H^+^, protons) into the gastric lumen in exchange for potassium ions (K^+^) in an ATP-driven process. This enzyme represents the final step in gastric hydrochloric acid secretion and constitutes therefore a target place for PPIs (Sachs, 2003). The structure of the gastric H^+^/K^+^-ATPase and the binding sites of PPIs are illustrated in Figure 2.

### 2.3 Pharmacological properties

PPIs cross biological membranes of parietal cells as prodrugs. PPIs are weak bases with a pKa between 3.8 and 4.5, which enables them to accumulate specifically in the acidic space of the secretory canaliculus of stimulated gastric parietal cells. The extremely acidic environment of these cells promotes protonation and transformation of PPI prodrugs into the active form. Following sequential protonation of the pyridine and benzimidazole moieties, PPIs are converted into sulfenamide intermediates (Shin et al., 2004; Sachs et al., 2006). Following protonation, sulfenamide intermediates of PPIs covalently bind to cysteine residues on the H^+^/K^+^-ATPase, primarily cysteine 813, leading to irreversible enzyme inhibition (Shin and Sachs, 2008; Shin et al., 2008). Additional binding to other cysteine residues varies among PPIs. Omeprazole and its S-enantiomer esomeprazole primarily form covalent bonds with cysteine 813 and 892 on the H^+^/K^+^-ATPase. Binding to cysteine 813, located within the luminal vestibule of the proton pump, results in effective inhibition of proton transport, whereas interaction with cysteine 892, positioned on the external luminal surface, may contribute to prolonged activity. Pantoprazole exhibits slower acid activation kinetics and binds predominantly to cysteine 813 or 822. The second domain is less accessible to reducing agents, which may contribute to more stable and less reversible inhibition of the pump. Lansoprazole shows relatively rapid activation and binds to cysteine 813 and/or 822 and additionally to cysteine 321. The accessibility of the latter within the luminal vestibule may facilitate reversible interactions under reducing conditions (Sachs et al., 2006). Rabeprazole initially binds within the transmembrane region containing cysteine 813 and/or cysteine 822. Following complete inhibition of the proton pump, rabeprazole continues to react with additional cysteine residues, including 321 and 892. This sequential binding pattern distinguishes rabeprazole from other PPIs and may contribute to differences in the stability of enzyme inhibition and the pharmacodynamic properties of this compound (Besancon et al., 1997).

The pharmacokinetic and pharmacodynamic properties of PPIs vary between individual drugs and can influence their clinical performance, onset of action, duration of acid suppression, and interaction potential. All PPIs have good oral bioavailability. For omeprazole, this parameter is relatively low after a single dose (about 35–40%), but may increase to around 65% with repeated administration and reduced gastric acid-related degradation (Cederberg et al., 1989; Andersson et al., 1990). For rabeprazole, absolute bioavailability upon oral administration is slightly higher and accounts for approximately 52% (Swan et al., 1999). Esomeprazole exhibits rapid and efficient absorption following oral administration, with a bioavailability ranging from 50 to 68% (Janiaczyk and Ogrodowczyk, 2021). Due to its superior pharmacokinetic properties, it demonstrates improved clinical efficacy, particularly in the management of GERD (Olbe et al., 2003). For the next two PPIs, pantoprazole and lansoprazole, consistently high bioavailability is observed (roughly 77 and 80–91%, respectively) (Stedman and Barclay, 2000).

Differences in cysteine binding among PPIs may account for the observed variations in duration of gastric acid inhibition. Although currently available benzimidazole-based PPIs share a short plasma half-life of approximately 1–2 h, their antisecretory effect persists (Gerloff et al., 1996; Radhofer-Welte, 1999). Due to the potential instability of covalent binding to the H^+^/K^+^-ATPase, the duration of gastric acid inhibition by PPIs is shorter than 54 h, which is estimated half-life of the enzyme. The estimated recovery half-times of gastric acid secretion in humans differ across PPIs, ranging from less than 15 h for lansoprazole to around 46 h for pantoprazole, which is closely related to the availability and strength of cysteine binding of proton pumps by PPIs (Katashima et al., 1998; Sachs, 2001; Shin and Sachs, 2002).

Cytochrome P450 (CYP) enzymes are a superfamily of heme-containing enzymes responsible for the phase I metabolism of a wide range of endogenous compounds and xenobiotics, including drugs. CYP enzymes are found predominantly in the liver and small intestine, with lower levels present in the kidneys, lungs, brain, and other tissues. In humans, CYP enzymes are grouped into families and subfamilies based on sequence homology, with CYP1, CYP2, and CYP3 representing the main drug-metabolizing families. The majority of human drug metabolism is mediated by following CYP isoenzymes: CYP1A2, CYP2C9, CYP2C19, CYP2D6, CYP2E1, and CYP3A4 (Blume et al., 2006). Among them, CYP2C19 and CYP3A4 are the most important isoenzymes involved in the metabolism of PPIs (Zanger and Schwab, 2013). For example, CYP2C19 is responsible for > 80% of the metabolism of omeprazole, lansoprazole and pantoprazole. A huge part of the interindividual variability in the pharmacokinetics of this group of drugs is associated with the CYP2C19 genotype of the treated patients. Phenotypic CYP2C19 activity is commonly classified into ultra-rapid, rapid, normal, intermediate, and poor, based on an individual’s underlying genotype. These categories reflect combinations of CYP2C19 alleles that determine enzyme function (El Rouby et al., 2018). Poor metabolizer phenotypes are observed more frequently in Asian and Oceanian populations compared with Caucasian-European and African populations, resulting in a higher proportion of individuals with reduced CYP2C19-mediated drug metabolism in these groups. In contrast, ultra-rapid metabolizers are more commonly found among individuals of Caucasian-European and African ancestry, where increased CYP2C19 activity leads to faster drug clearance (Xie et al., 1999; Desta et al., 2002; Klotz et al., 2004). Therefore, it is clear that clinical outcomes of the PPI therapy, as well as its adverse effects, might be partially determined by the patient’s inheritance.

The principal metabolic pathways of individual PPIs are summarized in Table 1.

**TABLE 1:** Pharmacological differences among proton pump inhibitors (PPIs).

PPI	Acid activation and principal H^+^/K^+^ -ATPase cysteine binding	Oral bioavailability	Plasma half-life	Major metabolic pathway/CYP2C19 dependence	Impact of CYP2C19 genotype	References
Omeprazole	Cys813 and Cys892	Approximately ∼35–40% after a single dose; may rise to ∼65% with repeated administration	∼1–2 h	Mainly CYP2C19 (> 80%), with additional CYP3A4 involvement	High	Shin et al., 2004; Sachs et al., 2006; Shin and Sachs, 2008; Shin et al., 2008; Stedman and Barclay, 2000; Blume et al., 2006; Zanger and Schwab, 2013; El Rouby et al., 2018.
Esomeprazole	Cys813 and Cys892	∼50–68%	∼1–2 h	Mainly by CYP2C19 and CYP3A4	Moderate to high	Shin et al., 2004; Sachs et al., 2006; Shin and Sachs, 2008; Shin et al., 2008; Olbe et al., 2003; Janiaczyk and Ogrodowczyk, 2021; Zanger and Schwab, 2013.
Pantoprazole	Cys813 and Cys822	∼77%	∼1–2 h	Mainly CYP2C19 (> 80%), with additional CYP3A4 involvement	High	Sachs et al., 2006; Katashima et al., 1998; Sachs, 2001; Shin and Sachs, 2002; Stedman and Barclay, 2000; Blume et al., 2006; Zanger and Schwab, 2013.
Lansoprazole	Cys813, Cys822, and Cys321	∼80–91%	∼1–2 h	Mainly CYP2C19 (> 80%), with additional CYP3A4 involvement	High	Sachs et al., 2006; Katashima et al., 1998; Sachs, 2001; Shin and Sachs, 2002; Stedman and Barclay, 2000; Blume et al., 2006; Zanger and Schwab, 2013.
Rabeprazole	Cys813 and Cys822; After complete pump inhibition, continues to react with Cys321 and Cys892	∼52%	∼1–2 h	Mainly through a non-enzymatic pathway; CYP2C19 contributes to a lesser extent	Low	Besancon et al., 1997; Swan et al., 1999; Blume et al., 2006; Zanger and Schwab, 2013; El Rouby et al., 2018.

CYP, cytochrome P450; GERD, gastroesophageal reflux disease; H + /K + -ATPase, gastric proton pump.

## 3 Clinical indications for proton pump inhibitors in acid-related diseases

The widespread use of PPIs in the treatment of acid-related gastrointestinal disorders results from their broad therapeutic efficacy, favorable pharmacokinetic profile, and lower incidence of adverse effects compared with other acid-suppressive drugs.

PPIs are successfully used for the short-term treatment of GERD and for the long-term prevention of its recurrence. They are also applied in the prevention and management of both erosive and non-erosive reflux esophagitis. Whether PPIs can delay the progression of esophageal intestinal metaplasia, known as Barrett’s esophagus, has not been conclusively demonstrated (Mössner, 2016). PPIs are frequently used in the acute and long-term management of eosinophilic esophagitis. They constitute also first-line therapy for the secondary prevention of gastroduodenal lesions associated with non-steroidal anti-inflammatory drugs (NSAIDs), including aspirin, as well as anticoagulant therapy. PPIs remain the treatment of choice in acute peptic ulcer disease, as well as in the management of peptic ulcer bleeding and the prevention of rebleeding following successful endoscopic hemostasis. They are also widely used for ulcer prophylaxis to reduce the risk of gastrointestinal bleeding in patients in intensive care units (Strand et al., 2016; Sloan and Katz, 2021). Although surgical excision of neuroendocrine tumors is the primary treatment for Zollinger-Ellison syndrome, almost all patients require PPI therapy to prevent acid-related complications resulting from hypergastrinemia and consequent gastric acid hypersecretion. However, data from large clinical studies remain limited due to the rarity of this condition (Strand et al., 2016).

The wide therapeutic spectrum, high efficacy, and broad availability of PPIs make them a cornerstone of acid-suppressive therapy. Their extensive range of indications across gastrointestinal and extra-gastrointestinal disorders has led to their routine use in everyday medical practice in both acute treatment and long-term preventive strategies.

## 4 Clinical indications for proton pump inhibitors in *Helicobacter pylori* infections

### 4.1 *Helicobacter pylori* infections

For many years, the highly acidic human stomach was believed to be a sterile environment. This paradigm changed in 1982 when Robin Warren and Barry Marshall isolated spiral bacteria from the gastric mucosa of patients suffering from gastric ailments. Their groundbreaking discovery was recognized with the Nobel Prize in Physiology or Medicine in 2005 (Wroblewski et al., 2010). Originally, this bacterium was known as *Campylobacter pylori*, but it was later renamed *Helicobacter pylori* following its reclassification (Goodwin, 1994).

*H. pylori* is a microaerophilic, Gram-negative, spiral-shaped bacterium that can persistently colonize the human gastric mucosa and plays a key role in the development of various gastroduodenal diseases (Yamaoka, 2010). Apart from inducing chronic gastritis and peptic ulcers, accumulating evidence from prospective studies had established a strong association between *H. pylori* infection and non-cardia gastric cancer, leading the World Health Organization to classify this bacterium as a Group I carcinogen in 1994 (International Agency for Research on Cancer, 1994). Subsequent research demonstrated its role in the development of gastric adenocarcinomas and gastric mucosa-associated lymphoid tissue (MALT) lymphomas, largely as a consequence of chronic inflammation induced by persistent infections (Kim et al., 2010).

*H. pylori* can be transmitted through several routes, including fecal-oral, oral-oral, and gastric-oral pathways. Current evidence indicates that the fecal-oral route is the predominant mode of transmission. Whereas the importance of oral-oral and gastro-oral transmission is not clear, these modes of transmission seem to gain significance during the colonization of the oral cavity by *H. pylori*, especially in patients with GERD. Nevertheless, although the oral cavity can serve as an additional reservoir for *H. pylori*, the stomach constitutes the primary site where this pathogen resides (Duan et al., 2023; Cuba et al., 2025).

**FIGURE 3:**
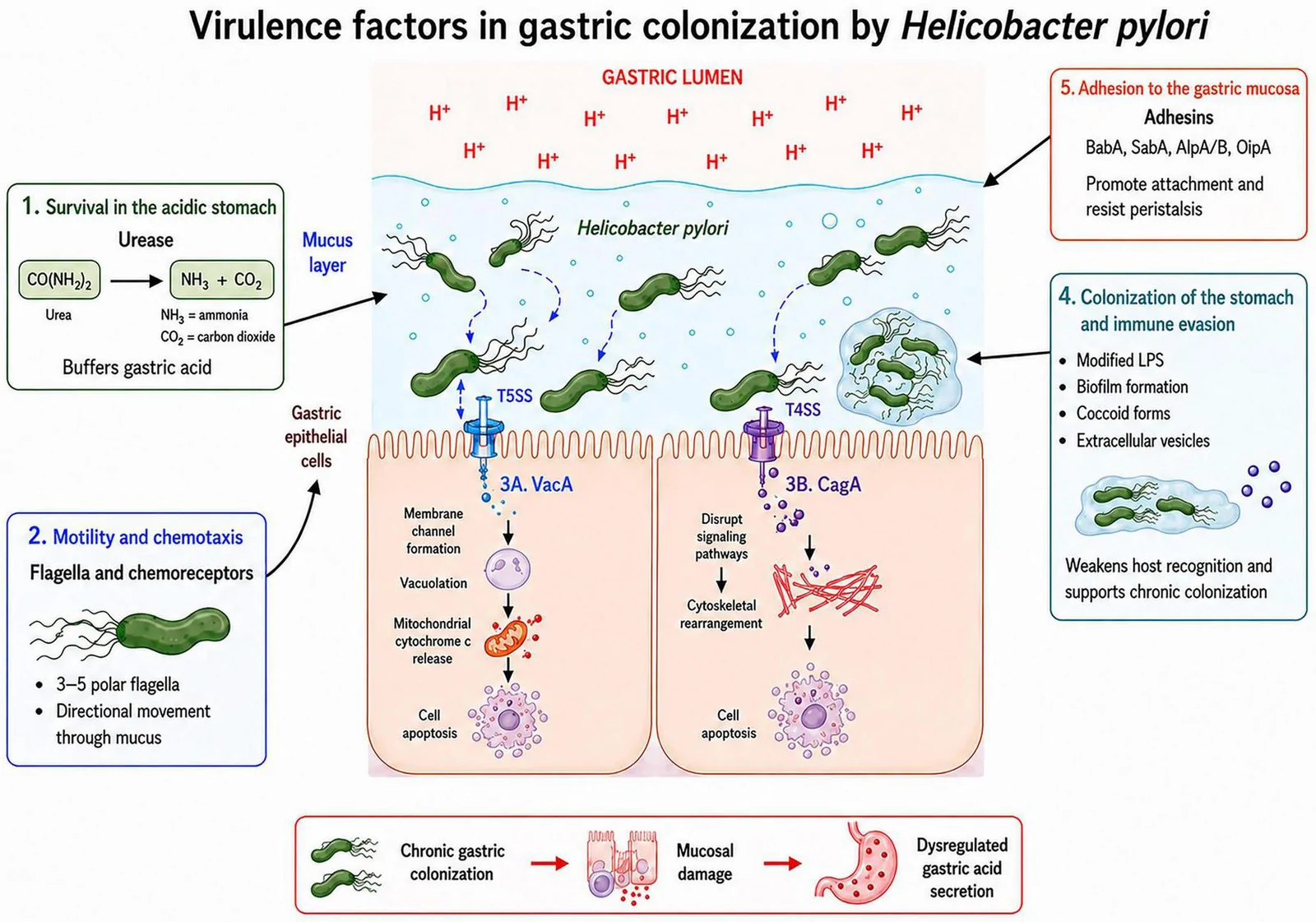
Key virulence factors essential for the successful colonization of the stomach by *Helicobacter pylori*. Abbreviations: AlpA/B, adherence-associated lipoprotein A/B; BabA, blood-group antigen binding adhesin; CagA, cytotoxin-associated protein A; LPS, lipopolysaccharide; OipA, outer inflammatory protein A; SabA, sialic acid-binding adhesin; T4SS, type IV secretion system; T5SS, type V secretion system; VacA, vacuolating cytotoxin A.

This bacterium is catalase- and oxidase-positive and equipped with 3–5 polar flagella that are used for its movement (Yamaoka, 2010). Among its virulence factors, urease is essential for survival and colonization of the acidic gastric environment. By hydrolyzing urea into ammonia and carbon dioxide, urease neutralizes gastric acid, facilitates bacterial persistence, and modulates host immune responses. Directional movement relative to the gastric mucosa is facilitated by flagella and a set of chemoreceptors that enable the detection of environmental chemical stimuli (Johnson and Ottemann, 2017). Subsequent stages of colonization of this organ involve strong adhesion, being mediated by a range of adhesion proteins [e.g., blood-group antigen binding adhesin (BabA), sialic acid-binding adhesin (SabA), adherence-associated lipoprotein A/B (AlpA/B), and outer inflammatory protein A (OipA)], all of which counteract the peristalsis of the digestive tract and the removal of these bacteria. Moreover, *H. pylori* lipopolysaccharide (LPS) is structurally modified, making it far less immunogenic than the LPS of other Gram-negative bacteria and resulting in a relatively weak host immune response (Matos et al., 2021). The ability of this microorganism to chronically colonize the stomach and evade the immune response is further enhanced by its capacity to form biofilms (Krzyżek et al., 2020), transform into coccoid forms (Krzyżek and Grande, 2020), or secrete extracellular vesicles (Chen et al., 2026; Figure 3).

In addition to its ability to evade the host’s response, *H. pylori* actively destroys the gastric mucosa and acquires nutrients obtained in this way. The two most important and best-studied lytic factors are vacuolating cytotoxin A (VacA) and cytotoxin-associated protein A (CagA). VacA is expelled to the local microenvironment via type V secretion system (T5SS) and induces cellular vacuolation, membrane-channel formation, and mitochondrial cytochrome c release, all of which lead to apoptosis and proinflammatory signaling. Furthermore, VacA suppresses T-cell activation and proliferation, stimulating therefore local immunosuppression (Foegeding et al., 2016). On the other hand, CagA is delivered into host cells via the type IV secretion system (T4SS), where it disrupts cellular signaling pathways, affects cell proliferation, motility, and apoptosis, and induces cytoskeletal rearrangements. These drastic changes in gastric cell physiology can in turn lead to the activation of oncogenic processes (Hatakeyama, 2017; Figure 3).

Multiple experimental and clinical studies have demonstrated that both asymptomatic and symptomatic *H. pylori* infections are associated with significant morphostructural and physiological changes in the stomach (Malfertheiner et al., 2023). These pathological changes contribute to dysregulation of gastric secretory physiology, making the control of gastric acidity a cornerstone of contemporary *H. pylori* eradication therapy.

### 4.2 PPIs as a central core of anti-*H. pylori* therapies

PPIs are an essential component of all *H. pylori* eradication regimens, as they neutralize gastric acidity, thereby creating conditions that enhance the efficacy and stability of the accompanying antibiotics (Malfertheiner et al., 2023). Given the gradual rise in *H. pylori* resistance over recent decades, triple or quadruple therapy regimens are currently employed. Current first-line regimens combine a PPI with two or three antimicrobials and are selected according to local *H. pylori* antibiotic resistance patterns. In regions with low clarithromycin resistance, triple therapy with a PPI, amoxicillin, and clarithromycin is advised, whereas in areas with high or unknown resistance to this antibiotic, quadruple therapy consisting of a PPI, metronidazole, tetracycline, and bismuth salts is recommended. If these prove ineffective, levofloxacin- or rifabutin-based treatment regimens are implemented (Malfertheiner et al., 2022).

The most commonly used PPIs in eradication regimens of *H. pylori* include omeprazole, esomeprazole, lansoprazole, pantoprazole, and rabeprazole (McNicholl et al., 2012). Among these, esomeprazole and rabeprazole may provide more consistent acid suppression and appear less affected by CYP2C19 genetic polymorphisms, which can influence treatment outcomes (Lima et al., 2021). The standard PPI doses used in *H. pylori* eradication therapy are administered twice daily and include omeprazole 20 mg, esomeprazole 20 mg, lansoprazole 30 mg, pantoprazole 40 mg, and rabeprazole 20 mg; however, various clinical trials are currently underway to determine the appropriate dosage of PPIs. In this context, preliminary data suggest that doubling the standard dose for some treatment regiments may enhance the therapeutic effect (the so-called, high-dose PPI therapies) (Pabón-Carrasco et al., 2023). These observations suggest that the role of PPI dose in *H. pylori* therapy may reflect additional antimicrobial mechanisms of PPIs that extend beyond their pH-buffering effects.

### 4.3 Direct anti-bacterial activity of PPIs

During the first two decades of research aimed at developing effective treatment regimens for *H. pylori* infections, considerable attention was devoted to the *in vitro* antimicrobial activity of PPIs. The first study, published by Iwahi et al. (1991), showed that lansoprazole exhibited greater antibacterial activity than omeprazole, with MIC values of 3.13–12.5 μg/mL and 12.5–50 μg/mL, respectively. These findings were confirmed by Nakao (1995), who demonstrated MIC ranges of 1.56–25 μg/mL for lansoprazole and 6.25–50 μg/mL for omeprazole. Subsequent studies consistently reported lower MIC values for lansoprazole than for omeprazole. Midolo et al. (1996) documented MIC ranges of 4–8 μg/mL for lansoprazole and 8–32 μg/mL for omeprazole, whereas Alarcón et al. (1998) reported ranges of 0.064–4 μg/mL and 4–32 μg/mL, respectively. Later studies expanded these observations to other PPIs. Rabeprazole showed the greatest *in vitro* activity, with MIC values ranging from 0.063 to 1 μg/mL, outperforming both lansoprazole (0.25–2 μg/mL) and omeprazole (0.5–16 μg/mL) (Kawakami et al., 2000). Esomeprazole also demonstrated potent antibacterial activity, with MIC values ranging from 8 to 32 μg/mL, whereas pantoprazole exhibited the weakest activity among the PPIs evaluated, with MIC values ranging from 25 to 100 μg/mL (Yu et al., 2017). In contrast, no standard agar- or broth-dilution MIC data are available for dexlansoprazole as it was developed later and was not included in the early antimicrobial screening studies (Table 2).

**TABLE 2:** Minimum inhibitory concentration (MIC) ranges of different proton pump inhibitors (PPIs) against *Helicobacter pylori*.

PPI	Reported MIC range (μ g/mL)	Reference reporting the MIC range
Omeprazole	12.5–50	Iwahi et al., 1991
	6.25–50	Nakao, 1995
	8–32	Midolo et al., 1996
	4–32	Alarcón et al., 1998
	6.25–25	Nakao and Malfertheiner, 1998
	0.5–16	Kawakami et al., 2000
	12.5–50	Yu et al., 2017
Esomeprazole	0.125–128	Yu et al., 2017
Pantoprazole	25–100	Nakao and Malfertheiner, 1998
	25–100	Yu et al., 2017
Lansoprazole	3.13–12.5	Iwahi et al., 1991
	1.56–25	Nakao, 1995
	4–8	Midolo et al., 1996
	0.064–4	Alarcón et al., 1998
	0.78–6.25	Nakao and Malfertheiner, 1998
	0.25–2	Kawakami et al., 2000
	3.13–12.5	Yu et al., 2017
Rabeprazole	0.063–1	Kawakami et al., 2000
	0.125–1	Soto et al., 2009
	0.16–1	Yu et al., 2017
Dexlansoprazole	No standard MIC data available	–

The ranges combine values reported across the cited studies. Lower MIC values indicate greater *in vitro* antibacterial activity. Differences between studies may reflect variation in *H. pylori* strains and susceptibility-testing methods.

In addition to the experiments determining MIC values of PPIs, some studies have also identified mechanisms governing the antibacterial activity of these compounds against *H. pylori*. Under acidic conditions, PPIs are converted into reactive sulfenamide derivatives capable of binding to bacterial proteins. This mechanism is considered a major contributor to the direct antibacterial activity of PPIs against *H. pylori*. By interacting with sulfhydryl groups in enzymes and other essential proteins, PPIs may impair key bacterial functions, including the induction of structural changes, inhibition of bacterial growth, and interference with enzymes, such as urease and ATPase (Nagata et al., 1993; Shin and Sachs, 2008). Although some studies indicate that the urease constitutes the primary target of PPIs (Nagata et al., 1993; Mirshahi et al., 1998; Saniee et al., 2016), others proved that PPIs maintain their growth-inhibitory activity even against urease-deficient *H. pylori* strains (Saniee et al., 2016). These findings suggest that PPIs have additional targets within bacterial cells. In line with this, some research proved for lansoprazole ability to inhibit *H. pylori* growth by disrupting key enzymes involved in energy metabolism and respiratory chain function (Nagata et al., 1995; Nakao and Malfertheiner, 1998). Recent studies by Kadkhodaei et al. (2020) have also shown that exposure of *H. pylori* to PPIs is accompanied by a range of changes in the fatty acid composition and membrane fluidity of the bacterium. Irrespectively of the PPIs and the microbial target affected, the extent of this effect depends on many factors, such as pH, oxygen concentration, drug concentration, and exposure time (Nagata et al., 1993, 1995; Tsuchiya et al., 1995; Midolo et al., 1996; Sjøstrøm et al., 1997; Nakao and Malfertheiner, 1998).

**FIGURE 4:**
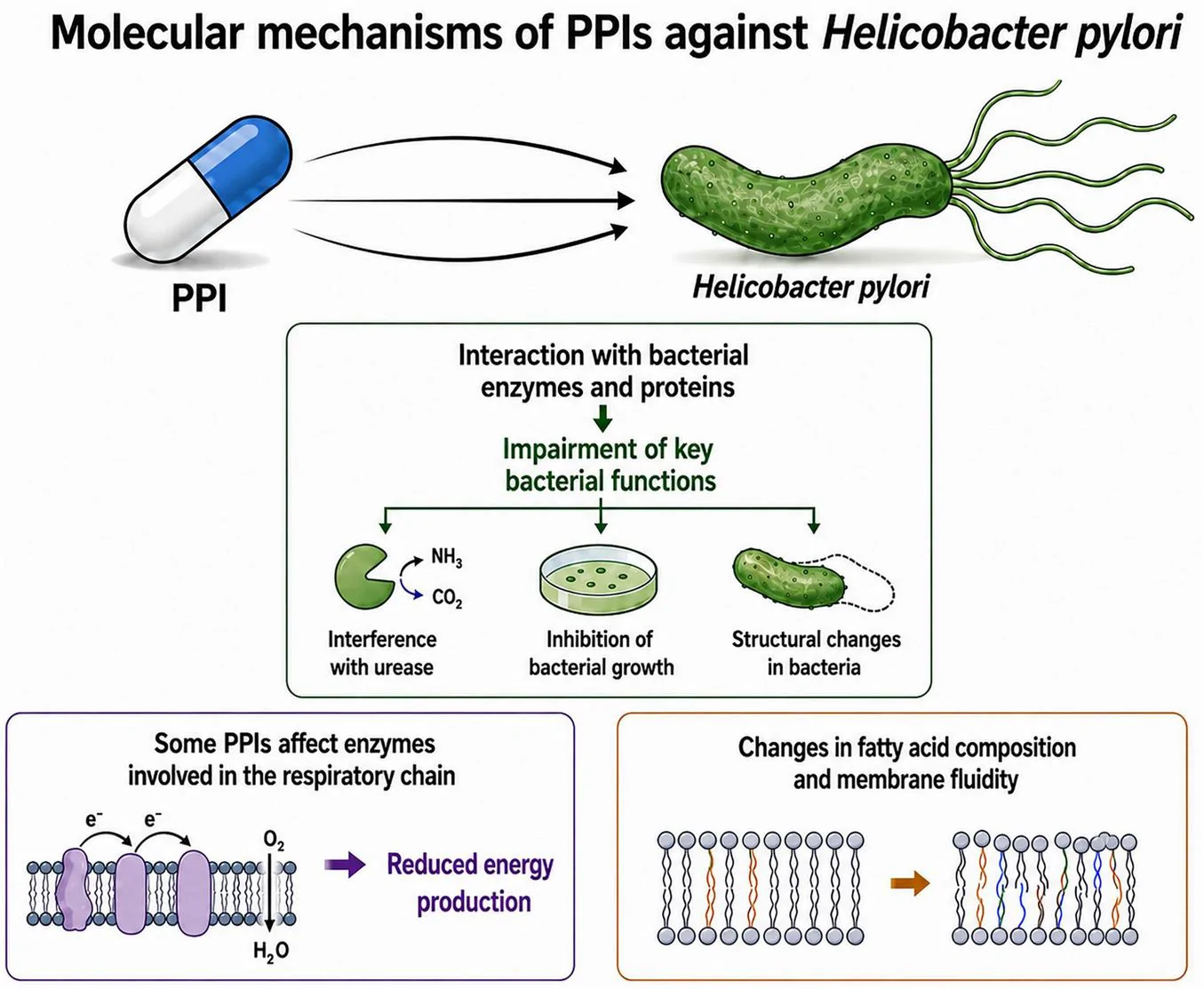
Molecular mechanism of the antibacterial action of proton pump inhibitors (PPIs) against *Helicobacter pylori*.

In summary, PPIs may inhibit *H. pylori* growth through multiple mechanisms, including disruption of bacterial metabolism, interference with virulence, and alterations in cell membrane integrity (Figure 4). Nevertheless, the precise contribution of these effects to the antibacterial activity of PPIs in *in vivo* conditions remains unclear.

### 4.4 Synergism of PPIs with antibiotics

Synergistic *in vitro* interactions between PPIs and antibiotics used in *H. pylori* treatment were demonstrated very early in the 1990s (Cederbrant et al., 1994; Nakao, 1995). In this context, the studies employed the widely renowned checkerboard assay and fractional inhibitory concentration index (FICI) analysis. First evidence for the direct interaction between PPIs and antibiotics was provided by Cederbrant et al. (1994), who demonstrated that the combination of omeprazole with clarithromycin produced predominantly additive interactions. These findings were further extended by Alarcón et al. (1998), who evaluated the interaction between omeprazole and clarithromycin or amoxicillin. The combination of omeprazole and clarithromycin demonstrated synergism against 51.5% of the strains and additive activity against 39.3% of them. In contrast, the combination of omeprazole and amoxicillin exhibited synergistic effect against only 3.0% of the strains, while additivity was observed in 60.6%. On the other hand, Nakao (1995) investigated the interactions between lansoprazole and 12 different antibiotics, including several not currently used for *H. pylori* eradication therapy. Most combinations exhibited additive effects; however, the strongest synergistic interactions were observed with the translation-inhibiting antibiotics clarithromycin and tetracycline, with synergism detected in 5 of the 18 strains tested for each antibiotic. To further elucidate this phenomenon, Malizia et al. (1998) investigated the interactions between omeprazole or lansoprazole and various macrolide antibiotics. All combinations demonstrated stimulatory interactions. The most pronounced synergistic effects were observed for the roxithromycin-lansoprazole, flurithromycin-omeprazole, and azithromycin-lansoprazole combinations, which exhibited synergistic activity against 82, 60, and 60% of *H. pylori* strains, respectively. After many years, Soto et al. (2009) set out to determine the interaction between the bactericidal antibiotics used in *H. pylori* therapy (metronidazole and amoxicillin) and the newest proton pump inhibitor–rabeprazole. It was observed that while these combinations often produced a synergistic effect (30–64%), a significant proportion of them were neutral or even antagonistic in nature.

Although the *in vitro* benefit of using PPIs in eradication therapies targeting *H. pylori* is well established, their antibiotic-potentiating effects remain incompletely understood. One of the reasons for this positive interaction is the pH-buffering capacity of these compounds. Current Maastricht recommendations emphasize that effective PPI-dependent acid suppression is crucial for successful eradication of *H. pylori.* By increasing gastric pH, PPIs enhance antibiotic efficacy and improve antimicrobial penetration into the gastric mucus layer. Additionally, by raising local pH of the stomach, these bacteria enter the replicative state and become more susceptible to antibiotics. At the same time, it should be noted that most studies evaluating the interaction between PPIs and antibiotics are conducted under neutral pH conditions, indicating that the nature of this interaction extends beyond the protective properties regarding antibiotics (Scott et al., 2016; Malfertheiner et al., 2022). In light of the molecular mechanism underlying the activity of PPIs against *H. pylori* proposed by Kadkhodaei et al. (2020), one possible explanation for the potentiation of antibiotic activity by PPIs is their ability to alter bacterial cell membrane permeability, thereby enhancing the intracellular uptake of co-administered antibiotics. To date, however, this mechanism remains hypothetical and has not been experimentally demonstrated. Another potential mechanism involves the ability of PPIs to inhibit the activity of efflux pumps, which constitute membrane proteins responsible for the non-specific extrusion of a wide range of drugs. Such inhibitory activity has been demonstrated in numerous microbial species (Vidaillac et al., 2007; Lake et al., 2023; Lutfi et al., 2025). Literature reports indicate that *H. pylori* also possesses multiple efflux pump systems that contribute to resistance against conventional antibiotics (Krzyżek, 2024). In line with this, PPIs may enhance antibiotic efficacy by suppressing the activity of these transporters also in case of *H. pylori*. Although this hypothesis is biologically plausible, it has not yet been validated experimentally.

**FIGURE 5:**
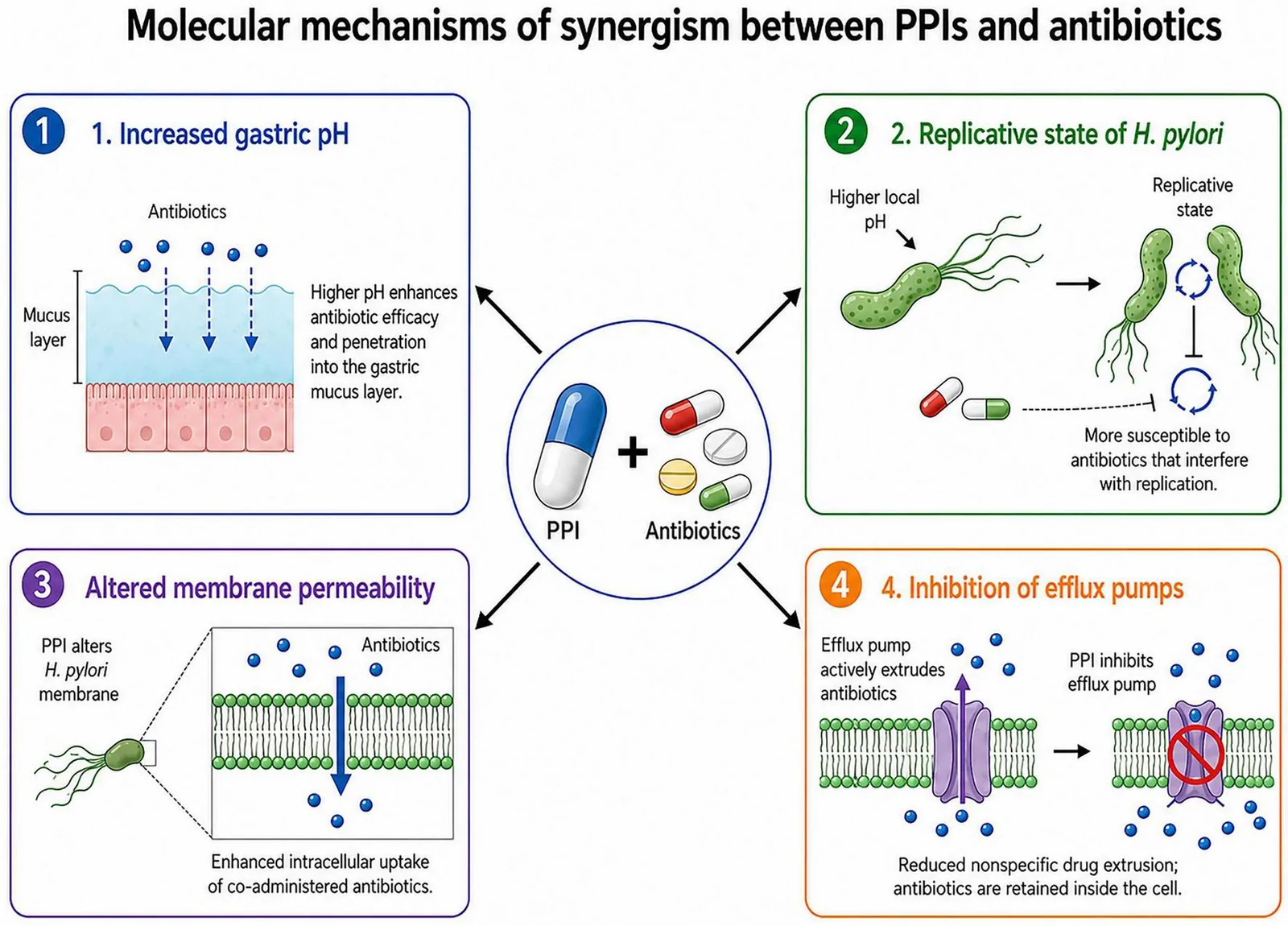
Molecular mechanism of the synergistic antibacterial action of proton pump inhibitors (PPIs) and antibiotics against *Helicobacter pylori*.

Collectively, these studies suggest that PPIs can in many cases potentiate the activity of antibiotics against *H. pylori*, although the mechanisms underlying these interactions remain poorly understood (Figure 5). Further *in vivo* studies are needed to clarify the clinical relevance of these mechanisms and their potential role in optimizing eradication regimens, particularly in the context of increasing antibiotic resistance.

## 5 Downsides of proton pump inhibitors: side effects, drug interactions, and overprescribing

### 5.1 Side effects

Although PPIs remain highly effective in the treatment of acid-related diseases, concerns regarding long-term therapy and potential adverse effects continue to increase. Current evidence suggests that, in patients with appropriate indications, the therapeutic benefits of PPIs generally outweigh potential risks. Therefore, PPIs should not be unnecessarily discontinued, although treatment duration and dosage should be limited to the minimum effective level (Ksiądzyna et al., 2015).

PPIs taken over a prolonged period of time lead to substantial changes in the structure and function of the stomach. The most consistent structural changes in the gastric mucosa are the development of parietal cell hyperplasia and hypertrophy. Morphological changes in gastric parietal cells, including cellular protrusion and swelling, have been observed after 3 months of continuous omeprazole therapy (Cats et al., 2000). Long-term PPI therapy may promote the development of fundic gland polyps (FGPs). FGPs occur 2–4 times more frequently in patients receiving long-term PPI therapy, with their prevalence increasing with treatment duration. Although FGPs have no proven malignant potential and do not require removal, the presence of numerous polyps may suggest an underlying hereditary syndrome, such as familial adenomatous polyposis (FAP) caused by germline *APC* mutations (Hongo and Fujimoto, 2010; Martin et al., 2016)

Gastric hydrochloric acid neutralizes ingested microorganisms; therefore, alterations in gut microbiota composition may occur due to an increase in gastric pH and a consequent reduction in the stomach’s antimicrobial activity. These changes may contribute to dysbiosis, diminished colonization resistance, and increased susceptibility to enteric infections caused by *Salmonella* spp., *Shigella* spp., pathogenic *E. coli* strains, or *Campylobacter* spp. (Bavishi and DuPont, 2011). Strong and prolonged acid inhibition might also lead to alterations in gut microbiota composition, especially when PPIs are co-administrated with antibiotics. This situation severely increases the risk of *C. difficile* infection and the development of antibiotic-associated diarrhea, pseudomembranous colitis, and toxic megacolon (Malfertheiner et al., 2017). Another circumstance associated with co-administrated of PPIs with antibiotics is an increased risk of gastric *Candida* spp. colonization. This occurs because the spectrum of antibiotics does not cover yeasts, whereas PPIs-related acid suppression stimulates fungal overgrowth in the upper gastrointestinal tract (Mottaghi et al., 2021). On the other hand, a decrease in stomach and small intestine acidity after repeated exposure to PPIs may promote the development of SIBO. Clinically, SIBO is frequently associated with chronic diarrhea and may result in malabsorption (Dukowicz et al., 2007; Malfertheiner et al., 2017).

Importantly, chronic use of high-dose PPIs can affect the absorption of vitamins and minerals, such as calcium, magnesium, and vitamin B_12_, since acid facilitates assimilation and ionization of less soluble forms of dietary cations and release of food-bound vitamin B_12_. These changes in the vitamin and mineral economy may in turn translate into an increased risk of different severe illnesses and disorders, such as osteoporosis and hip fractures, cardiac arrhythmias, neuromuscular disturbances, peripheral neuropathy, megaloblastic anemia, and possibly dementia (Malfertheiner et al., 2017). The above-mentioned associations warrant careful consideration due to their potential clinical relevance; however, their interpretation remains controversial, as subsequent analyses indicate that these findings may be explained by confounding factors and methodological limitations rather than a direct causal relationship. Despite inconsistent evidence, the U.S. Food and Drug Administration (FDA) introduced warnings regarding fracture risk associated with prolonged or high-dose PPIs use (Mössner, 2016; Strand et al., 2016; Haastrup et al., 2018). A list presenting potential side effects of PPI therapies is presented in Table 3.

**TABLE 3:** Potential adverse effects associated with long-term proton pump inhibitor (PPI) therapy.

Adverse effect	Clinical consequences	References
Alterations in gastric morphostructure	• Parietal cell hyperplasia and hypertrophy • Chronic atrophic gastritis • Development of fundic gland polyps	Cats et al., 2000; Hongo and Fujimoto, 2010; Martin et al., 2016
Microbiota alterations	• Increased susceptibility to enteric infections caused by *Salmonella* spp., *Shigella* spp., pathogenic *Escherichia coli*, and *Campylobacter* spp. • Increased risk of *Clostridioides difficile* infections including antibiotic-associated diarrhea, pseudomembranous colitis, and toxic megacolon • Fungal overgrowth and increased gastric *Candida* spp. colonization • Small intestinal bacterial overgrowth	Bavishi and DuPont, 2011; Dukowicz et al., 2007; Malfertheiner et al., 2017; Mottaghi et al., 2021
Vitamin and mineral malabsorption	Reduced absorption of: • Calcium • Magnesium • Vitamin B_12_	Malfertheiner et al., 2017
Other potential systemic consequences	• Osteoporosis and hip fractures; • Cardiac arrhythmias • Neuromuscular disturbances • Peripheral neuropathy • Megaloblastic anemia • Possible association with dementia	Malfertheiner et al., 2017; Mössner, 2016; Strand et al., 2016; Haastrup et al., 2018

### 5.2 Drug interactions

As noted in the previous paragraphs, almost all PPIs are metabolized mainly in the liver by CYP enzymes, which may lead to drug to drug interactions. Most cytochrome P450-mediated drug interactions arise from competitive inhibition at the enzyme’s active site, as co-administered substrates compete for the limited metabolic capacity of CYP isoforms (Zanger and Schwab, 2013). Because many commonly prescribed drugs are metabolized by CYP2C19 and CYP3A4, PPIs may increase plasma concentrations of co-administered drugs (Li et al., 2004). In line with this, omeprazole, esomeprazole, lansoprazole, and dexlansoprazole can affect the activity of these two enzymes, potentially altering the metabolism of other drugs (Stedman and Barclay, 2000; El Rouby et al., 2018; Andersson et al., 2001). In contrast, pantoprazole has a relatively low affinity for CYP enzymes and is partly metabolized via sulfotransferase pathways, resulting in a low risk of clinically significant drug interactions. Rabeprazole differs from most other PPIs because it is primarily metabolized through a non-enzymatic pathway to rabeprazole thioether, and to a lesser extent undergoes hepatic metabolism mediated by CYP2C19. As a result, rabeprazole is less affected by genetic polymorphisms of CYP2C19 (Bakheit et al., 2021).

One of the most significant examples of the influence of PPIs on interactions with other drugs is clopidogrel. Clopidogrel is an oral antiplatelet agent used in the prevention of atherothrombotic events, including myocardial infarction and ischemic stroke, particularly in patients with acute coronary syndrome or after percutaneous coronary intervention (Malfertheiner et al., 2017). Because clopidogrel requires CYP2C19 for activation, omeprazole and esomeprazole may reduce its activation by inhibiting this enzyme. Although pharmacokinetic studies confirm reduced formation of active metabolites, clinical data are inconsistent (Bavishi and Hirai, 2026). Evidence further indicates that coadministration of omeprazole or esomeprazole reduces the exposure to the active metabolite of clopidogrel and impairs platelets inhibition, which may be associated with an increased risk of composite ischemic end points, all-cause mortality, non-fatal myocardial infarction, stroke, revascularization, and stent thrombosis in patients on clopidogrel (Wedemeyer and Blume, 2014; Malfertheiner et al., 2017). Interestingly, rabeprazole appears to have a lower potential for interaction with clopidogrel compared with omeprazole, likely due to its weaker inhibition of CYP2C19-mediated clopidogrel activation. Therefore, based on current pharmacodynamic evidence, omeprazole and its stereoisomer esomeprazole are generally avoided in patients receiving clopidogrel therapy, in favor of alternative PPIs such as rabeprazole, lansoprazole, dexlansoprazole, or pantoprazole (Funck-Brentano et al., 2013; Strand et al., 2016).

Among other clinically relevant drug to drug interactions involving PPIs, particular attention should be given to those involving warfarin, which is an oral vitamin K antagonist used for the prevention and treatment of thromboembolic disorders, and tacrolimus, which is an immunosuppressant used for prevention of organ transplant rejection (Christians et al., 2002; Holbrook et al., 2005). Such situation is strictly associated with their narrow therapeutic windows and the risk of clinically relevant changes in drug exposure mediated by cytochrome P450-dependent metabolic pathways. Omeprazole and esomeprazole may reduce the clearance of warfarin through inhibition of CYP2C19, whereas pantoprazole, lansoprazole and rabeprazole have not been associated with clinically relevant metabolic interactions with warfarin (Blume et al., 2006). Increased clearance of warfarin results in reduced anticoagulant effect leading to a higher risk of thromboembolic events (Holbrook et al., 2005). On the other hand, among PPIs, lansoprazole has been most consistently associated with increased tacrolimus exposure, likely through reduced tacrolimus clearance. This interaction may result in elevated tacrolimus blood concentrations and an increased risk of tacrolimus-related toxicity. Therefore, therapeutic drug monitoring is recommended when PPIs, particularly lansoprazole, are co-administered with tacrolimus (Christians et al., 2002; Blume et al., 2006).

Another clinically relevant issue is the effect of PPIs on gastric pH, which can reduce the absorption of drugs that require an acidic environment, such as ketoconazole, itraconazole, indinavir, L-thyroxin, and iron salts (Stedman and Barclay, 2000). Consequently, concomitant PPI therapy may compromise the effectiveness of selected antifungal and antiviral treatments, hormonal therapies, and supplement-based interventions (Blume et al., 2006).

**FIGURE 6:**
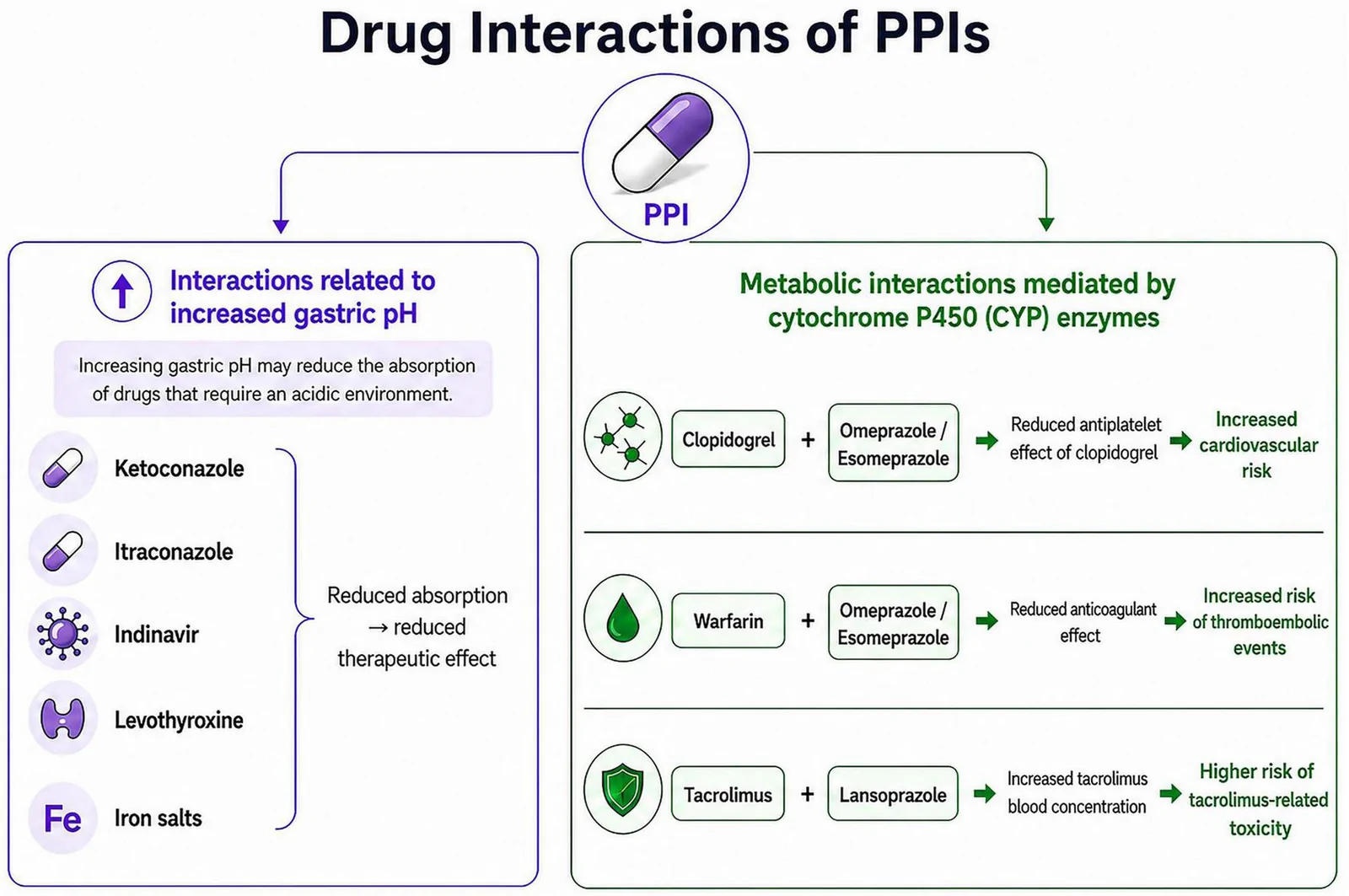
A schematic diagram illustrating the interactions between proton pump inhibitors (PPIs) and specific drugs, along with a description of side effects.

The above data clearly demonstrate the ability of PPIs to influence a number of physiological characteristics of patients and the therapies they receive (Figure 6). Despite the growing evidence regarding clinically relevant drug to drug interactions associated with PPIs, awareness of these interactions remains insufficient, while the number of PPI prescriptions without clear medical indications continues to be substantial.

### 5.3 Overprescribing

Given their well-established efficacy, favorable safety profile, and good tolerability, PPIs are widely used worldwide. Overprescribing of these medications is frequently observed in both primary care and hospital practice. According to ClinCalc DrugStats data, PPIs remain one of the most frequently prescribed drug classes in the United States, with approximately 86 million prescriptions issued in 2023 (Kane, 2025). In Asia, national data highlight significant PPI utilization with high absolute values observed in China, meanwhile a sharp increase was seen in South Korea after ranitidine withdrawal (approximately 3929 doses/100,000/day) (Dutta et al., 2023; Zeng et al., 2023; Choi et al., 2025). The situation is similar in Europe. Notably, more than 35 million PPI prescriptions were recorded in the United Kingdom in 2022–2023 (Andrawes et al., 2025). Additionally, during the 12-month study period from November 2020 to October 2021, approximately 2.87 billion PPI tablets were sold in Germany, corresponding to an estimated consumption of approximately 34 tablets per German resident. Notably, 97% of them were sold by prescription and over 59% of PPI users were aged above 60 (Plehhova et al., 2024). Although comprehensive nationwide data on PPI utilization and inappropriate prescribing in Poland are limited, Polish authors have also recognized PPI overuse as an arising clinical problem (Ksiądzyna et al., 2015).

Studies suggest that 25–70% of PPI prescriptions lack an appropriate indication (Andrawes et al., 2025). Evidence suggests that PPIs are frequently initiated upon hospital admission in elderly patients as a “gastroprotection” to avoid any potential prosecutions against physicians in charge for neglecting medical care (Heidelbaugh and Inadomi, 2006; Eid et al., 2010). Studies show that over half of non-intensive care unit patients were given acid-suppressive therapy, but 65% of prescriptions were not justified. In addition, PPIs overuse in the clinical setting is often the result of incorrect stress ulcer prophylaxis in non-intensive care unit patients, and failure to discontinue this therapy prior to the hospital discharge. Over 55% of the patients receiving any form of prophylactic gastric acid inhibition were discharged on the therapy (Nardino et al., 2000; Ksiądzyna et al., 2015). In a large UK primary care cohort, approximately 25% patients continued PPI therapy for over a year. Many of these did that without their initial indications for long-term use (Othman et al., 2016).

The widespread use of PPIs has raised concerns within the scientific community and among regulatory authorities, as an increasing body of observational evidence has linked long-term PPI use to a range of potential adverse outcomes. At the same time, growing awareness of the frequent inappropriate initiation and prolonged use of PPIs has further highlighted the need for their more judicious prescribing.

## 6 Potassium-competitive acid blockers as an alternative for PPIs

Although PPIs have revolutionized the management of acid-related disorders owing to their efficacy, favorable pharmacokinetic profile, and overall safety, their delayed onset of action, interindividual variability in response, and incomplete acid suppression have highlighted the need for alternative therapeutic approaches.

The development of potassium-competitive acid blockers (P-CABs) began in the 1980s as an alternative approach for PPIs. SCH_2_8080, the first imidazopyridine-derived P-CAB, showed rapid and effective inhibition of gastric acid secretion, but its clinical development was halted because of hepatotoxicity associated with long-term treatment. Subsequent research led to the synthesis of several structurally diverse P-CABs, including vonoprazan, linaprazan, soraprazan, and revaprazan. Although several P-CABs have been developed, only a limited number have reached clinical practice owing to safety concerns and other developmental challenges. Revaprazan became the first P-CAB approved for clinical use in South Korea, whereas vonoprazan was introduced in Japan in 2015 and subsequently approved in several Asian countries (Domingues et al., 2023). In 2023, vonoprazan (Voquezna^®^) received FDA approval through the New Drug Application pathway for the treatment of acid-related disorders and as part of *H. pylori* eradication therapy (U.S. Food and Drug Administration, 2023). Currently, vonoprazan, tegoprazan, fexuprazan, and keverprazan are approved in several Asian countries, whereas the availability of P-CABs in Europe still remains limited (Scarpignato and Hunt, 2024). Among these agents, vonoprazan is the only P-CAB currently undergoing clinical evaluation in Europe for the treatment of acid-related disorders, including gastroesophageal reflux disease and *H. pylori* infection, with the aim of supporting future regulatory approval (European Medicines Agency, 2024). Accordingly, this section focuses primarily on vonoprazan, which currently has the most robust clinical evidence and is considered the P-CAB with the greatest potential for future clinical use in Europe.

As mentioned in the previous part of this article, proton pump is a transmembrane protein, transferring equal amounts of hydrogen and potassium ions along with passive movement of chloride ions to maintain an acidic gastric environment (so called H^+^/K^+^-ATPase). Both P-CABs and PPIs block the activity of this protein, however, through different mechanisms. P-CABs selectively and reversibly compete with luminal potassium ions for a binding site in H^+^/K^+^-ATPase, resulting in rapid, reversible inhibition of gastric acid secretion. This mechanism contrasts with that of PPIs, which inhibit the enzyme through covalent binding to the α-subunit of H^+^/K^+^-ATPase (Scarpignato and Hunt, 2024). Unlike currently available PPIs, which are all substituted benzimidazole derivatives, P-CABs represent a distinct, heterogenous group of compounds with completely different chemical structures. For example, vonoprazan is a pyrrole-based compound that lacks the imidazopyridine ring found in several other P-CABs, such as fexuprazan, whereas tegoprazan, revaprazan, and linaprazan are composed of distinct chemical structures (Scarpignato and Hunt, 2024; Qiao and Li, 2025).

P-CABs are lipophilic weak bases with high pKa values and remain stable under acidic conditions. This combination of properties allows them to concentrate in low pH compartments. Moreover, all P-CABs are able to bind proton pump regardless of whether the enzyme is in its active or inactive conformation. Most of these newer compounds exhibit rapid and effective antisecretory activity. Among currently available P-CABs, vonoprazan represents a major advance in acid suppression, providing more potent and sustained inhibition of the gastric proton pump compared with traditional PPIs and several other acid-suppressive agents (Scarpignato and Hunt, 2021). Vonoprazan is metabolized primarily by CYP3A4, with minor contributions from CYP2 enzymes (including CYP2C19), sulfation, and glucuronidation. Consequently, its pharmacokinetics are largely unaffected by CYP2C19 genetic polymorphisms. Nevertheless, potential drug to drug interactions mediated by CYP enzyme inhibition should still be considered, including the possibility of reduced antiplatelet activity of clopidogrel (Yamasaki et al., 2017). Following oral administration, vonoprazan is rapidly absorbed, reaching peak plasma concentrations within 1–3 h (Shirley, 2024). Its pharmacokinetics are approximately dose-proportional, with steady-state concentrations achieved after 3–4 days, and are not significantly affected by food intake (Jenkins et al., 2015; Mulford et al., 2022). Clinical data indicate that, compared with PPIs, vonoprazan provides rapid, potent, and sustained acid suppression, maintaining intragastric pH above 4 for most of the 24-h period after repeated administration (Jenkins et al., 2015).

The efficacy of vonoprazan-based eradication therapy has been confirmed in several meta-analyses. An early meta-analysis demonstrated that vonoprazan-based triple therapy was superior to conventional PPI-based triple therapy in first-line *H. pylori* eradication, while maintaining a comparable safety and tolerability profile (Jung et al., 2017). More recent systematic reviews have confirmed its efficacy in both first- and second-line treatment strategies, with particular advantages observed in patients infected with clarithromycin-resistant strains (Jung et al., 2017; Benito et al., 2024). Additionally, studies evaluating high-dose dual therapy with vonoprazan and amoxicillin suggest that effective eradication can be achieved without use of clarithromycin, supporting the potential role of simplified P-CAB-based regimens (Furuta et al., 2020; Suzuki et al., 2020). Table 4 provides a quantitative comparison of vonoprazan- and PPI-based therapies based on the latest meta-analysis by Lv et al. (2026).

**TABLE 4:** Quantitative comparison of *Helicobacter pylori* eradication outcomes with PPI-based and vonoprazan-based regimens based on the meta-analysis of Lv et al. (2026).

Outcome	Vonoprazan-based therapy	PPI-based therapy	Effect
ITT eradication	88.0%	82.7%	RR 1.05 (95% CI 1.00–1.10), ***P* = 0.04**
PP eradication	92.3%	87.3%	RR 1.04 (95% CI 1.01–1.08), ***P* = 0.02**
Adverse events	8.6%	10.6%	RR 0.78 (95% CI 0.59–1.04), *P* = 0.09
Compliance	97.1%	95.6%	RR 1.02 (95% CI 0.99–1.05), *P* = 0.22

Systematic review and meta-analysis of four randomized controlled trials involving 1,807 treatment-naive patients. The comparison included vonoprazan plus high-dose amoxicillin dual therapy and PPI plus high-dose amoxicillin dual therapy. CI, confidence interval; HDDT, high-dose dual therapy; ITT, intention-to-treat; *P*, probability value; PP, per-protocol; PPI, proton pump inhibitor; RR, risk ratio; VPZ, vonoprazan. Bolded *P* values indicate a statistically significant difference between the groups.

Current guidelines, including the Maastricht VI/Florence consensus report, recognize P-CAB-containing therapies as effective alternatives to PPI-based regimens in *H. pylori* eradication, particularly in regions with increasing antimicrobial resistance. The Maastricht Consensus emphasizes that successful *H. pylori* eradication requires maintaining an intragastric pH of approximately 6–7, a target that conventional PPIs often fail to sustain over a 24-h period (Malfertheiner et al., 2022). Currently, FDA approved vonoprazan for the treatment of erosive esophagitis, maintenance of healing of erosive esophagitis, relief of heartburn associated with erosive and non-erosive GERD, and as a component of dual or triple therapy for *H. pylori* eradication in adults (U.S. Food and Drug Administration, 2023). Beyond established indications, ongoing research suggests potential future applications of P-CABs in idiopathic peptic ulcer disease, prevention of delayed bleeding after endoscopic submucosal dissection, and non-erosive reflux disease (Scarpignato and Hunt, 2024).

Although PPIs remain the cornerstone of acid-suppressive therapy, emerging evidence suggests that P-CABs may represent a promising alternative for the management of acid-related disorders and *H. pylori* infection. Their rapid and sustained acid suppression has shown encouraging clinical efficacy; however, current evidence is insufficient to support the routine replacement of PPIs. Further large-scale, long-term, and multiethnic studies are needed to establish the efficacy, safety, and optimal role of P-CABs in clinical practice. The differences between the mechanisms of action of PPIs and P-CABs are described in Table 5.

**TABLE 5:** Pharmacological and clinical comparison of proton pump inhibitors (PPIs) and potassium-competitive acid blockers (P-CABs).

Parameter	Proton pump inhibitors (PPIs)	Potassium-competitive acid blockers (P-CABs)	References
Chemical class	Substituted benzimidazole derivatives	Structurally heterogeneous compounds; vonoprazan is pyrrole-based, whereas other P-CABs have distinct chemical scaffolds	Scarpignato and Hunt, 2024; Qiao and Li, 2025
Site of action	Luminal surface of the gastric parietal-cell H^+^/K^+^-ATPase	Potassium-binding region of the gastric parietal-cell H^+^/K^+^-ATPase	Scarpignato and Hunt, 2024
Mechanism of action	Covalently bind to cysteine residues on the alpha-subunit of H^+^/K^+^-ATPase, producing irreversible inhibition	Selectively and reversibly compete with luminal K^+^ for binding to H^+^/K^+^-ATPase, producing reversible inhibition	Scarpignato and Hunt, 2024
Requirement for acid activation	Yes	No	Scarpignato and Hunt, 2021; Scarpignato and Hunt, 2024
Pump conformation requirement	Primarily inhibit active proton pumps	Inhibit both active and inactive proton pumps	Scarpignato and Hunt, 2024
Metabolism	Mainly hepatic metabolism through CYP2C19 and CYP3A4, with the relative contribution varying among individual PPIs	Vonoprazan is metabolized mainly by CYP3A4, with additional CYP2-family, sulfation, and glucuronidation pathways	Yamasaki et al., 2017
Onset of action	Delayed	Rapid	Jenkins et al., 2015; Scarpignato and Hunt, 2021
Potency and duration of acid suppression	Effective, but acid suppression may be incomplete or variable over 24 h	More potent and sustained; vonoprazan maintains intragastric pH > 4 for most of 24 h	Jenkins et al., 2015; Scarpignato and Hunt, 2021
Effect of food and timing of administration	Affected by food intake	Not significantly affected by food intake	Jenkins et al., 2015; Mulford et al., 2022
Drug-interaction potential	Potential CYP-mediated interactions, particularly for agents that inhibit or compete for CYP2C19 and CYP3A4	CYP-mediated interactions remain possible	Yamasaki et al., 2017
Role in *H. pylori* eradication	Standard component of conventional eradication regimens	May improve eradication outcomes; recommended in selected regimens but not yet routinely used	Jung et al., 2017; Furuta et al., 2020; Suzuki et al., 2020; Malfertheiner et al., 2022; Benito et al., 2024

CYP, cytochrome P450; GERD, gastroesophageal reflux disease; H^+^/K^+^-ATPase, gastric proton pump.

## 7 Brief recommendations for clinicians and scientists

To emphasize the importance of key aspects outlined in the current review, five practical recommendations for clinicians and researchers are presented below:

1. Regular evaluation of the ongoing need for PPI therapy, with continuation reserved for patients with a clear evidence-based indication and consideration of deprescribing when long-term treatment is no longer warranted.
2. Monitoring for potential long-term adverse effects of PPI therapy, including alterations in the gut microbiota, SIBO, and deficiencies of essential vitamins and minerals, such as vitamin B_12_, magnesium, and calcium.
3. Potential drug-drug interactions should be assessed before initiating or continuing PPI therapy, particularly in patients receiving multiple medications, with special attention to interactions involving omeprazole and clopidogrel, as well as certain PPIs and tacrolimus or warfarin.
4. In patients with an inadequate response to PPI therapy, CYP2C19 metabolic variability should be considered and therefore dose adjustment, switching to a PPI that is less dependent on CYP2C19 metabolism, or using an alternative acid-suppressive therapy may improve treatment outcomes, particularly in rapid and ultrarapid metabolizers.
5. Where available and recommended by the current Maastricht guidelines, P-CABs should be considered as an alternative to PPIs, particularly for *H. pylori* eradication.

## 8 Conclusion

The introduction of PPIs has revolutionized the management of acid-related disorders by providing effective and sustained suppression of gastric acid secretion, which has significantly improved the treatment of numerous gastrointestinal conditions. In line with this, PPIs remain an essential component of modern acid-suppressive treatment and, due to their unique pharmacological properties, continue to play a central role in *H. pylori* eradication strategies. Over time, however, their widespread availability and extensive clinical use have contributed to concerns regarding inappropriate prescribing and the potential consequences of long-term therapy. Although P-CABs represent a promising alternative with encouraging results in *H. pylori* eradication, further studies are required to determine their long-term safety, efficacy, and potential role in replacing or complementing PPIs in clinical practice.

## 9 Literature search strategy

A structured literature search was conducted in PubMed/MEDLINE, Scopus, Web of Science, and Google Scholar, covering publications available from database inception to July 2026. The search strategy was based on keyword combinations using the Boolean operators AND and OR of the following terms: “proton pump inhibitors,” “PPI,” “PPIs,” “omeprazole,” “esomeprazole,” “lansoprazole,” “dexlansoprazole,” “pantoprazole,” “rabeprazole,” “*Helicobacter pylori*,” “antibacterial activity,” “acid suppression,” “CYP2C19,” “drug interactions,” “adverse effects,” “overprescribing,” “potassium-competitive acid blockers,” “P-CAB,” “P-CABs,” and “vonoprazan.” Original research articles, clinical trials, systematic reviews, meta-analyses, clinical guidelines, and relevant narrative reviews published in English were considered, whereas duplicate records, conference abstracts lacking sufficient data, articles unavailable in full text, and publications not directly related to the scope of this review were excluded.
